# Relocating a pediatric hospital: Does antimicrobial resistance change?

**DOI:** 10.1186/s13104-020-05065-7

**Published:** 2020-05-13

**Authors:** Annika Schönfeld, Rudolf Ascherl, Stefanie Petzold-Quinque, Norman Lippmann, Arne C. Rodloff, Wieland Kiess

**Affiliations:** 1 Hospital for Children and Adolescents, University Hospital Leipzig, Liebigstraße 20a, 04103 Leipzig, Germany; 2 Institute of Medical Microbiology and Epidemiology of Infectious Diseases, University Hospital Leipzig, Liebigstraße 21, 04103 Leipzig, Germany

**Keywords:** Health facility moving, Drug resistance, Microbial, Hospitals, Pediatric

## Abstract

**Objective:**

Analyze the changes in antimicrobial drug resistance patterns due to hospital relocation. To this end, we conducted a retrospective analysis of microbiological results, especially minimum inhibitory concentrations (MIC) of all isolates from blood, urine and bronchial secretions, in our pediatric university hospital before and after moving to a new building.

**Results:**

While the number of tests done did not change, the fraction of those positive increased, more MICs were determined and certain microbes (*A. baumannii*, *E. faecalis*, *Klebsiella* spp. and *P. mirabilis*) were detected more frequently. Most changes in MICs occurred in *E. faecium* (increases in 8 antimicrobials, decreases only in linezolid and gentamicin). For imipenem and aminopenicillins the MICs commonly rose after relocation, the opposite is true for gentamicin and trimethoprim/sulfamethoxazole. The other factors that alter by moving a hospital such as changes in medical personnel or case severity cannot be corrected for, but using MICs we are able to provide insights into changes down to the individual antimicrobial drug and even small changes usually undetectable to the common categorical reporting of resistance.

## Introduction

Antimicrobial drug resistance (AMR) is surging: All around the world it increases morbidity and mortality putting strain on already tight health-care budgets [1, 2].

The influence buildings have on the relationship of humans and microbes is best exemplified by resemblance of microbiota in men and his edifices [3]. Even facilities located closely to one another harbor a different variety of bacteria [4]. Hourigan reported marked changes in resistance genes in a microbiome study on relocating a neonatal intensive-care unit (NICU). More AMR genes were found in the new NICU with single rooms as opposed to the old shared-space facility [5].

Under this pretense, it seems safe to assume AMR is a spacial phenomenon: Resistance patterns are so different from one location to another that clinical course and treatments might be diverging significantly. E.g. the paradigm of cutting down on antibiotic use to curtail AMR while true for Europe is not holding up on a global scale [6]. Models of AMR showed that there are patterns within cooperating health-facilities and AMR is easily spread by transfer of patients to naive collectives [7]. The dynamics of AMR and thus ways to influence them are mostly taking place on a local level [8]. The size of the smallest unit and their distance that can be expected to be different in AMR is not known. Examining microbiological tests before and after moving a department might give insights into this. Moving changes the amount of space and number of patients per room, potential cross-contamination from shared facilities, and other infrastructural factors like water supply or sewerage. But not only are such relocations rare, they are seldom studied, AMR even less so [9–13].

The relocation of our pediatric university hospital gave us an opportunity to examine AMR before and after relocation.

## Main text

### Methods

#### Patients and setting

Since its foundation in 1891 Leipzig’s pediatric university hospital had remained in an individual complex of edifices. It abandoned these premises to move into a new building adjacent to the rest of the university hospital in August 2007. We retrospectively examined all inpatient microbial tests in combination with the patient’s demographic data and diagnoses from 2000 until 2012 to describe changes after the relocation.

#### Microbiology

Identification of bacterial species was done by analytical profile indexing (API ID, bioMérieux, Marcy-l’Étoile, France) until replaced by a MALDI-TOF-based method (VITEK MS, bioMérieux, Marcy-l’Étoile, France) on 2012-01-01.

Minimum inhibitory concentrations (MIC) were measured as described by ISO 20776-1: Cultures suspended to a density of McFaland standard 0.5 were mixed with equal volumes of serial dilutions of antimicrobials (0.125 to 512 mg/L) in microtiter plates. The MIC is the lowest concentration inhibiting clouding of the culture by bacterial growth [14]. MICs of isolates from all blood, urine and tracheal secretion cultures obtained from 2000 until 2012 were included for in-depth analysis. Due to indispositions in our university’s laboratory department MICs obtained in 2010 were not available for study. To reduce misrepresentation from multiple sampling only one isolate with identical resistance profiles per admission was included.

#### Data analysis

Data were queried and extracted from the findings data base of the Institute of Microbiology. Analyses were done in the ‘R’ software environment [15] using the ‘plyr’ package [16]. To compare two samples of quantitative values Student’s *t* test was used. When any of its constraints were not met, Wilcoxon’s rank-sum test took its place instead. Categorical samples were put to the χ^2^-test. We rejected the null hypothesis when the canonical p < α = 0.05 was met.

### Results

#### General characteristics of the hospital and microbiological sampling

General annual characteristics of the hospital and microbiological sampling in the years before (2000–2006) and after (2008–2012) the relocation were summarized in Table 1. The total number of microbiological tests did not change significantly, neither did the share returning positive. We observed some differences within certain individual sample types: While their numbers were not at variance, there were less positive CSF cultures (5.2% vs. 3.6%, W = 31, p = 0.030) and more positive urine cultures (21% vs. 32%, W = 1, p = 0.005). There were less rectal swabs (112 vs. 24, W = 31, p = 0.028), but more came back positive (22% vs. 40%, W = 3, p = 0.018).

**Table 1:** General characteristics of the hospital and microbiological sampling therein

	Before relocation	After relocation	
Hospital size (beds)			
ICU (beds)	21	22	
NICU (beds)	29	30	
Patients treated per year (n)^a^	6306 (6206–6440)	6721 (6674–6751)	W = 0, p = 0.036
Case mix index^a^	1.044 (1.032–1.070)	1.226 (1.185–1.342)	W = 0, p = 0.036
Microbiological samples (n), positive (%)	4548 (4415–4942) 15.1% (14.6–16.3%)	4506 (4456–4994) 17.7% (16.9–18.4%)	W = 16, p = 0.8763 W = 7, p = 0.1061
By type (total n, % positive)^+^			
Blood	1463 (1435.5–1479) 5.75% (5.32–6.98%)	1574 (1540–1600) 5.9% (5.13–6.3%)	W = 7, p = 0.1038 W = 20, p = 0.7551
CSF	507 (474.5–527.5) 5.23% (4.72–5.94%)	464 (440–465) 3.57% (2.5–3.81%)	W = 27, p = 0.149 W = 31, p = 0.030
Urine	666 (623.5–727) 20.7% (19.8–23.9%)	740 (645–805) 31.6% (31.5–32.9%)	W = 14, p = 0.639 W = 1, p = 0.005
Rectal swab	112 (106–265.5) 21.5% (17.8–28.1%)	24 (22–47) 40% (37.8–50%)	W = 31.5, p = 0.028 W = 3, p = 0.018
Stool	1039 (960.5–1070) 6.83% (6.3–7.28%)	1049 (1038–1117) 6.94% (6.91–11%)	W = 11, p = 0.343 W = 11, p = 0.268
Throat swab	607 (568–819) 41.7% (34.5–46%)	587 (540–788) 45.3% (44.8–49.4%)	W = 19, p = 0.876 W = 9, p = 0.202
Tracheal secretion	136 (133–145.5) 51.77% (50.47–52.57%)	123 (121–123) 62.7% (56.9–63.7%)	W = 29, p = 0.193 W = 8, p = 0.149

The year of the relocation (2007) was excluded and the calendar years 2000 to 2006 (“before relocation”) and 2008 to 2012 (“after relocation”) were compared

^a^Data are only available from 2004 through 2012, + seldomly done types are excluded

#### MIC of isolates

The frequencies of species for which MICs were determined are depicted in Additional file 1: Figure S1. An increase between 2000 to 2006 and 2008 to 2012 has been seen in *P. mirabilis* (t(6.24 = − 6.475), p < 0.001), *A. baumannii* (t(8.97) = − 3.31, p = 0.012), *E. faecalis* (t(7.59) = − 8.21, p < 0.001), *E. coli* (t(7.03) = − 4.51, p = 0.003), *K. oxytoca* (t(3.67) = − 3.87, p = 0.021) and *K. pneumoniae* (t(3.96) = − 3.99, p = 0.017).

To weigh the differences in MICs, we split the MIC data of individual blood, tracheal secretion and urine isolates along the month the relocation took place (August 2007). This left us with two samples: before (2000-01-01–2007-07-31) and after the relocation (2007-09-01–2012-12-31). Their general characteristics are outlined in Table 2, the exact changes for each species and antiinfective drug are detailed in Additional file 2: Table S1 and summarized in Table 3.

**Table 2:** Study cohort characteristics. Data before relocation are until 2007-08-01 and after are later than 2007-08-31

	Before relocation	After relocation	
No. of cultures^a^	33,100	23,779	
Positive	5119 (15.5%)	4448 (18.7%)	Χ(1) = 73.43, p < 0.001
MIC available	2692	1550	Χ(1) = 117.44, p < 0.001
Blood	604 (22.4%)	226 (14.6%)	Χ(1) = 26.04, p < 0.001
Urine	1396 (51.9%)	1029 (66.4%)	Χ(1) = 22.35, p < 0.001
Respiratory	692 (25.7%)	295 (19.0%)	Χ(1) = 15.22, p < 0.001
No. of patients (n)	1092	852	
Age (a)	1.46 (0.388–6.34)	1.67 (0.546–5.51)	t(3355) = 0.72, p = 0.47
Sex (% male)	50.7%	48.8%	Χ(1) = 1.3516, p = 0.245

**Table 3:** Summary of all significant differences in MICs before (2000-01-01–2007-08-01) and after the relocation (2007-08-31–2012-12-31) are displayed as either increase, ↑, or decrease, ↓; → denotes that no significant change has been demonstrated

	Penicillin	Ampicillin/Sulbactam	Piperacillin/Tazobactam	Cefuroxime	Cefotaxime	Meropenem	Imipenem	Gentamicin	Vancomycin	Teicoplanin
*Staphylococcus hominis*	→	→	→	→	→	→	↑	→	→	→
*Staphylococcus haemolyticus*	→	→	→	→	→	→	→	→	→	↑
*Staphylococcus epidermidis*	→	↑	→	↑	↑	↑	↑	→	→	→
*Staphylococcus aureus*	→	→	→	↑	↑	↑	↑	↓	→	↓
*Streptococcus pneumoniae*	→	↑		↑	→		↑			
*Streptococcus agalactiae*	→	→		→	→	→	↑		↑	→
*Streptococcus viridans*	↑			↑				→		
*Enterococcus faecalis*	↑	→	→				→	↓	→	↓
*Enterococcus faecium*	→	→	↑				↑	↓	↑	↑
*Enterobacter cloacae*	→	→	→	→	↓	→	→	→		
*Escherichia coli*	↓	↑	→	→	→	↑	↑	→		
*Haemophilus influenzae*	↑	↑		→	↑					
*Haemophilus parainfluenzae*	→	→		→	→		→			
*Klebsiella pneumoniae*	→	→	→	→	↓	↑	→	↓		
*Klebsiella oxytoca*	→	↑	→	→	→	→	↑	↑		
*Acinetobacter baumannii*	→	→	↑	→	↑	↑	↓	→		
*Proteus mirabilis*	→	→	↓	→	↑	→	→	→		
*Serratia marcescens*	→	→	↓	→	↓	↑	↑	→		
*Pseudomonas aeruginosa*	→	→	↑		→	↑	→	→		

	Ciprofloxacin	Levofloxacin	Moxifloxacin	Clindamycin	Linezolid	Rifampicin	Cotrimoxazole	Ampicillin	Ceftazidime	Colistin
*Staphylococcus hominis*	→	→	→	→	→	↓	→	→		
*Staphylococcus haemolyticus*	→	→	→	→	→	↓	↓			
*Staphylococcus epidermidis*	→	→	→	↓	→	↓	↓	→		
*Staphylococcus aureus*	→	↑	→	↓	→	↑	↑	→		
*Streptococcus pneumoniae*	→		→	→			→	↑		
*Streptococcus agalactiae*	→	→	→	→	→	→	→	→		
*Streptococcus viridans*			→	↓				↑		
*Enterococcus faecalis*	→	↑	↑	↓	↓	↓	↓	↑		
*Enterococcus faecium*	↑	↑	↑	→	↓	→	→	↑		
*Enterobacter cloacae*	→	↑	→				↓	→	→	→
*Escherichia coli*	↑	↑	↑				↓	↑	↑	→
*Haemophilus influenzae*	→							↑		
*Haemophilus parainfluenzae*	→			→			→	→		
*Klebsiella pneumoniae*	→	↓	→				↓	→	→	
*Klebsiella oxytoca*	→	→	→				→	↑	→	
*Acinetobacter baumannii*	→	→	→				↓	↑	↑	→
*Proteus mirabilis*	↓	↓	↓				↓	→	→	→
*Serratia marcescens*	↑	↓	→				↓	→	→	→
*Pseudomonas aeruginosa*	→	→	→				↓		↑	→

All comparisons were done as Wilcoxon tests if both samples contained at least 5 values

1135 (60%) of individual patients had 1 isolate, 95% had 6 or less. Of 1445 (77%) Patients one sample was sent in, 5 or less were sent in for 95%. Of the total 3360 positive cultures 2577 (77%) had one isolate, 97% had 2 or less.

The highest number of significant changes in MIC were seen in *E. faecium* (antimicrobials showing an increase in MIC, 2 with a decrease). All but linezolid and gentamicin rose after relocation. For *A. baumannii* all significant changes in MICs were higher with the exception of trimethoprim/sulfamethoxazole.

In all cases of significant differences for imipenem and ampicillin (with and without sulbactam) the MICs rose after the relocation, the opposite is true for gentamicin and trimethoprim/sulfamethoxazole.

### Discussion

Apart from a decrease in rectal swabs, the number of microbiological tests did not change. Less CSF tests came back positive, but more urines and rectal swabs.

We saw increases in the detection frequencies of some bugs, among them *A. baumannii* and *E. faecalis* that are notoriously difficult to treat. For the first we saw increases in MIC for many of the substances tested. Gentamicin and trimethoprim/sulfamethoxazole generally decreased in MICs, whereas MICs of imipenem and ampicillin commonly increased. Special note should be taken of opposite trends within substance groups like in carbapenems for *A. baumannii.*

There are several factors affected by the relocation apart from moving closer to the adult wards of our hospital and thus sharing some facilities with them: the number of patients per room, and the number of patients each caregiver attended to. Our study cohort covers vastly different patient collectives from septic neonates to adolescents with minor illnesses. On average, cases became more complex exemplified by a hike in case-mix index from 1.02 in 2004, when diagnosis related groups were introduced, to 1.43 in 2013. This might also be the reason why we see more microbiological tests returning positive and more MICs being determined. The relatively long time period also allows for factors other than the building to influence AMR: Different medical personnel and guidelines over a period of 12 years transform isolation and treatment strategies.

This is the first study comparing changes in MIC down to the individual substance before and after relocation of a hospital. The few comparable publications used aggregated data: Individual antimicrobials are grouped into classes and the proportion of resistant isolates is reported rather than MICs [17, 18].

Arndt compared the share of resistant isolates on short and long-term for the relocation of an adult ICU: The moving-related resistance rates were in the same direction as the long-term trends and increased in *Enterococci* and *E. coli* [10]. We can corroborate this by the frequent increases in MICs we see in these pathogens, but the high degree of depth of our data unearths changes for the positive otherwise unnoticed: E.g. in both *Ee. faecium* (p = 0.0017) and *faecalis* (p = 0.0006) MICs of linezolid fell significantly.

To delineate relocation-related differences from any general underlying trends we would need local data to compare our MIC to. These are not available. Nationwide German “Antibiotika-Resistenz-Surveillance” (antibiotic resistance surveillance) for example started only in 2008 and reports resistance only categorically.

The European Antimicrobial Resistance Surveillance Network (EARS-Net) of the ECDC only has few different species and antimicrobial classes on record, even less so in our study epoch. For E. coli they follow the same trends. In *Ee. faecalis* and *faecium* ECDC has not seen the increase in aminoglycoside susceptibility we see for Gentamicin. Aminopenicillin resistance fell in the EARS-Net data where we saw a hike.

Logan examined changes in *A. baumannii* resistance 1999 to 2012 in the USA and found significant trends with increases for carbapenems and cephalosporins [19]. In this publication the proportion of isolates showing resistance for at least one of the drugs in a given group were compared over time. Concurrently in our study, MIC rose in all cephalosporins over a similar epoch, but in carbapenems results were mixed.

While we are unable to correct for general trends in AMR or the many other influence factors, the present study also has some strengths: The use of MICs down to the individual drug enables us to notice subtle differences normally lost only looking at the categories of “resistant”, “intermediate”, or “susceptible”. Definition of these categories also change over time, a fact often overlooked when different epochs are compared to one another. This (1) gives us the chance to choose another antimicrobial drug from the same group otherwise thought to be ineffective, and (2) can help gain insights into general trends that indicate borderline or future sensibility justifying hope for treating AMR with the tools already available.

## Limitations

- Our data predate most registries of antimicrobial resistance making comparison difficult.
- We were unable to delineate the effects on moving from other influence factors.

## Supplementary information

**Additional file 1: Figure S1.** Stacked plot of the absolute number of bacterial isolates per species per year. The thick vertical line indicates the relocation date.

**Additional file 2: Table S1.** MICs of isolates from before (January 2000 through July 2007) and after relocation (September 2007 through December 2012).

## Data Availability

The datasets analysed during the current study are not publicly available due to privacy restrictions.

## Abbreviations

AMR: Antimicrobial drug resistance
CSF: Cerebrospinal fluid
MIC: Minimum inhibiting concentration
NICU: Neonatal intensive care unit
