# Supplementary figures and images for: Relocating a pediatric hospital: Does antimicrobial resistance change?
Source: BMC Res Notes. 2020 May 13;13:242. doi: 10.1186/s13104-020-05065-7 (PMC7218827; doi:10.1186/s13104-020-05065-7)

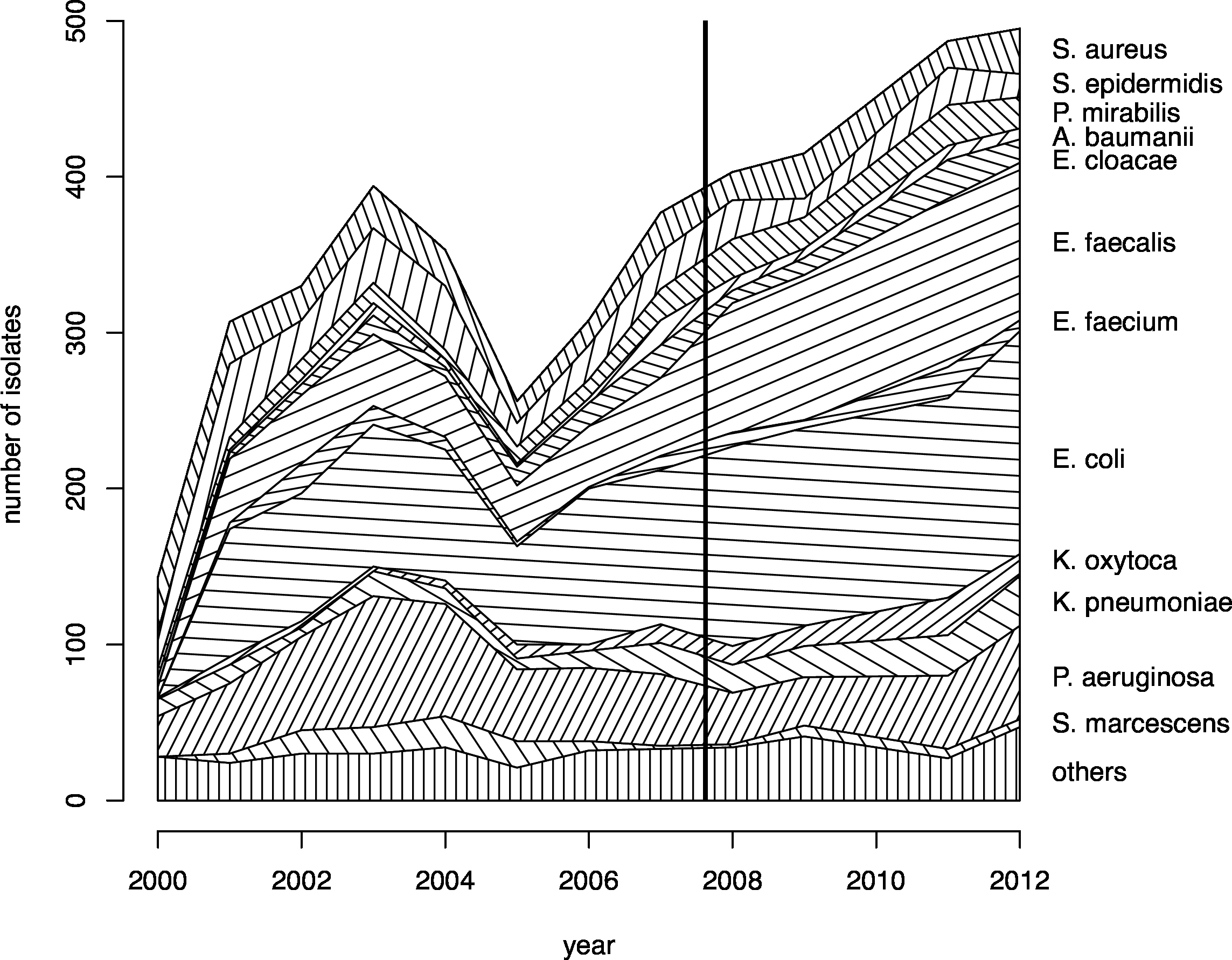

Supplement: Supplementary file 1 — Additional file 1: Figure S1. Stacked plot of the absolute number of bacterial isolates per species per year. The thick vertical line indicates the relocation date. [file 13104_2020_5065_MOESM1_ESM.jpg]
