# Supplementary material for: Relocating a pediatric hospital: Does antimicrobial resistance change?
Source: BMC Res Notes. 2020 May 13;13:242. doi: 10.1186/s13104-020-05065-7 (PMC7218827; doi:10.1186/s13104-020-05065-7)
Supplement: Supplementary file 2 — Additional file 2: Table S1. MICs of isolates from before (January 2000 through July 2007) and after relocation (September 2007 through December 2012). [file 13104_2020_5065_MOESM2_ESM.docx]

Table S1: MICs of all isolate samples (n>5) from before (January 2000 through July 2007) and after the relocation (September 2007 thorugh December 2012). MICs are displayed as median (IQR).

| isolate | relocation | Penicillin | Ampicillin | Ampicillin/Sulbactam | Piperacillin/Tazobactam | Cefuroxim |
| --- | --- | --- | --- | --- | --- | --- |
| Staphylococcus hominis | before | 2 (0.25 - 8, n=37) p=0.1977 | 1 (0.25 - 4, n=13) p=0.1111 | 0.5 (0.25 - 1, n=43) p=0.3224 | 0.5 (0.5 - 4, n=9) p=0.4186 | 1 (0.5 - 2, n=43) p=0.6921 |
|  | after | 4 (0.5 - 8, n=21) | 4 (3 - 24, n=7) | 1 (0.25 - 2, n=22) | 2 (1 - 4, n=22) | 1.5 (0.625 - 2, n=22) |
| Staphylococcus haemolyticus | before | 8 (8 - 8, n=31) p=0.8307 |  | 16 (2 - 32, n=31) p=0.2535 | 8 (8 - 16, n=10) p=0.2759 | 32 (7 - 32, n=32) p=0.2922 |
|  | after | 8 (8 - 8, n=14) |  | 32 (4 - 32, n=15) | 128 (6 - 128, n=15) | 32 (16 - 32, n=15) |
| Staphylococcus epidermidis | before | 8 (2 - 8, n=220) p=0.6315 | 8 (2 - 28, n=106) p=0.0609 | 1 (0.25 - 2, n=219) p=4e-04 | 1 (0.5 - 2, n=86) p=0.2623 | 4 (1 - 8, n=219) p=0.0096 |
|  | after | 8 (0.5 - 8, n=81) | 4 (0.5 - 16, n=39) | 4 (0.5 - 8, n=85) | 2 (0.5 - 8, n=82) | 8 (1 - 16, n=85) |
| Staphylococcus aureus | before | 2 (0.25 - 8, n=179) p=0.4027 | 4 (1 - 20, n=104) p=0.4253 | 0.25 (0.25 - 0.875, n=178) p=0.0827 | 1 (0.5 - 2, n=88) p=0.4312 | 1 (1 - 1, n=176) p=0 |
|  | after | 2 (0.1095 - 8, n=92) | 4 (0.25 - 8, n=35) | 0.25 (0.25 - 1, n=88) | 1 (0.5 - 1.25, n=88) | 1 (1 - 2, n=89) |
| Streptococcus pneumoniae | before | 0.031 (0.031 - 0.063, n=44) p=0.7933 | 0.125 (0.125 - 0.125, n=13) p=0.0283 | 0.125 (0.125 - 0.125, n=10) p=0.0428 |  | 0.125 (0.125 - 0.125, n=38) p=7e-04 |
|  | after | 0.031 (0.031 - 0.063, n=22) | 0.25 (0.125 - 0.25, n=10) | 0.25 (0.125 - 0.25, n=9) |  | 0.1875 (0.125 - 0.25, n=22) |
| Streptococcus agalactiae | before | 0.031 (0.031 - 0.031, n=8) p=0.1374 | 0.25 (0.1875 - 0.25, n=7) p=0.0972 | 0.25 (0.25 - 0.25, n=7) p=0.1392 |  | 0.125 (0.125 - 0.125, n=8) p=0.0582 |
|  | after | 0.031 (0.031 - 0.039, n=20) | 0.25 (0.25 - 0.25, n=10) | 0.25 (0.25 - 0.25, n=18) |  | 0.25 (0.125 - 0.25, n=21) |
| Streptococcus viridans | before | 0.063 (0.031 - 0.125, n=45) p=0.029 | 0.25 (0.125 - 0.25, n=35) p=0.0032 |  |  | 0.25 (0.125 - 0.5, n=39) p=0.03 |
|  | after | 0.5625 (0.063 - 1.75, n=14) | 1 (0.25 - 4, n=13) |  |  | 0.375 (0.25 - 3.5, n=14) |
| Enterococcus faecalis | before | 4 (2 - 4, n=232) p=0.4973 | 2 (1 - 2, n=131) p=0.0035 | 1 (1 - 2, n=282) p=0.9168 | 4 (4 - 4, n=97) p=0.1732 |  |
|  | after | 4 (2 - 4, n=371) | 2 (2 - 4, n=136) | 1 (1 - 2, n=388) | 4 (4 - 4, n=383) |  |
| Enterococcus faecium | before | 8 (8 - 8, n=48) p=0.8289 | 48 (32 - 64, n=14) p=0.0243 | 64 (32 - 64, n=51) p=0.1124 | 128 (128 - 128, n=14) p=0.0261 |  |
|  | after | 8 (8 - 8, n=35) | 64 (64 - 64, n=12) | 64 (64 - 64, n=39) | 128 (128 - 128, n=39) |  |
| Enterobacter cloacae | before |  | 64 (32 - 64, n=65) p=0.3428 | 64 (8 - 64, n=65) p=0.6813 | 4 (2 - 32, n=63) p=0.1681 | 16 (8 - 32, n=65) p=0.6322 |
|  | after |  | 64 (32 - 64, n=70) | 48 (16 - 64, n=70) | 4 (2 - 16, n=70) | 32 (8 - 32, n=69) |
| Escherichia coli | before |  | 8 (4 - 64, n=554) p=0 | 2 (1 - 32, n=553) p=0 | 2 (1 - 2, n=548) p=0.437 | 4 (4 - 4, n=553) p=0.0928 |
|  | after |  | 16 (4 - 64, n=555) | 4 (2 - 64, n=548) | 2 (1 - 2, n=547) | 4 (4 - 8, n=553) |
| Haemophilus influenzae | before |  | 0.25 (0.25 - 0.4375, n=26) p=0.0365 | 0.25 (0.25 - 0.25, n=26) p=0.0317 |  | 0.5 (0.25 - 1, n=26) p=0.4465 |
|  | after |  | 0.5 (0.25 - 0.5, n=21) | 0.375 (0.25 - 0.5, n=18) |  | 0.5 (0.5 - 1, n=22) |
| Haemophilus parainfluenzae | before | 0.25 (0.25 - 0.25, n=5) p=0.0203 | 0.25 (0.25 - 0.25, n=10) p=0.1076 | 0.25 (0.25 - 0.25, n=10) p=0.0665 |  | 0.5 (0.3125 - 0.5, n=10) p=0.074 |
|  | after | 0.5 (0.25 - 0.75, n=11) | 0.25 (0.25 - 1, n=20) | 0.5 (0.25 - 0.5, n=16) |  | 0.5 (0.4375 - 1, n=20) |
| Klebsiella pneumoniae | before |  | 32 (32 - 64, n=83) p=0.082 | 4 (2 - 16, n=83) p=0.9007 | 4 (2 - 8, n=83) p=0.109 | 2 (2 - 8, n=83) p=0.0652 |
|  | after |  | 32 (32 - 64, n=101) | 4 (2 - 8, n=101) | 2 (2 - 4, n=101) | 2 (2 - 4, n=101) |
| Klebsiella oxytoca | before |  | 32 (32 - 64, n=38) p=0.0025 | 3 (1 - 16, n=38) p=0.0052 | 2 (2 - 4, n=38) p=0.4823 | 4 (2 - 8, n=38) p=0.6897 |
|  | after |  | 64 (32 - 64, n=64) | 8 (4 - 32, n=64) | 2 (1 - 4, n=63) | 3 (2 - 8, n=66) |
| Acinetobacter baumanii | before |  | 16 (16 - 32, n=25) p=0.0233 | 0.25 (0.25 - 0.25, n=40) p=0.7858 | 0.5 (0.5 - 5, n=40) p=0.009 | 32 (16 - 32, n=25) p=0.143 |
|  | after |  | 32 (24 - 32, n=27) | 0.25 (0.25 - 0.25, n=30) | 8 (0.5 - 16, n=30) | 32 (32 - 32, n=28) |
| Proteus mirabilis | before |  | 2 (2 - 32, n=74) p=0.246 | 2 (1 - 2, n=73) p=0.0651 | 0.5 (0.5 - 1, n=74) p=0.0025 | 1 (1 - 2, n=74) p=0.9028 |
|  | after |  | 2 (2 - 64, n=102) | 2 (1 - 2, n=102) | 0.5 (0.5 - 0.5, n=102) | 1 (1 - 2, n=103) |
| Serratia marcescens | before |  | 64 (32 - 64, n=80) p=0.7944 | 64 (32 - 64, n=80) p=0.2229 | 16 (2 - 32, n=75) p=0 | 32 (32 - 32, n=80) p=0.5639 |
|  | after |  | 64 (56 - 64, n=20) | 64 (64 - 64, n=19) | 2 (1.5 - 2, n=19) | 32 (32 - 32, n=20) |
| Pseudomonas aeruginosa | before |  |  | 64 (64 - 64, n=66) p=0.0534 | 4 (4 - 8, n=397) p=0 |  |
|  | after |  |  | 64 (64 - 64, n=183) | 8 (4 - 16, n=185) |  |

| isolate | relocation | Cefotaxim | Ceftazidim | Meropenem | Imipenem | Gentamicin |
| --- | --- | --- | --- | --- | --- | --- |
| Staphylococcus hominis | before | 4 (1 - 8, n=43) p=0.5558 |  | 1 (0.125 - 2, n=43) p=0.3034 | 0.063 (0.063 - 0.125, n=43) p=3e-04 | 0.125 (0.125 - 4, n=43) p=0.3402 |
|  | after | 4 (1 - 8, n=22) |  | 2 (0.125 - 4, n=22) | 0.125 (0.125 - 0.40625, n=22) | 0.125 (0.125 - 1, n=22) |
| Staphylococcus haemolyticus | before | 32 (6 - 32, n=31) p=0.2349 |  | 8 (1 - 32, n=32) p=0.8888 | 0.25 (0.063 - 16, n=31) p=0.2999 | 512 (8 - 512, n=31) p=1 |
|  | after | 16 (16 - 16, n=15) |  | 8 (2 - 24, n=15) | 0.5 (0.125 - 20, n=15) | 512 (258 - 512, n=15) |
| Staphylococcus epidermidis | before | 8 (4 - 8, n=219) p=0.0323 |  | 1 (0.5 - 4, n=218) p=0.0126 | 0.125 (0.063 - 1, n=219) p=0 | 0.5 (0.125 - 512, n=220) p=0.4038 |
|  | after | 8 (2.5 - 16, n=82) |  | 2 (0.5 - 8, n=82) | 0.5 (0.125 - 8, n=86) | 8 (0.125 - 512, n=85) |
| Staphylococcus aureus | before | 2 (1 - 2, n=178) p=0 |  | 0.125 (0.063 - 0.125, n=176) p=0 | 0.063 (0.063 - 0.063, n=179) p=0 | 0.5 (0.25 - 0.5, n=179) p=0 |
|  | after | 2 (2 - 2, n=90) |  | 0.125 (0.125 - 0.125, n=88) | 0.125 (0.125 - 0.125, n=88) | 0.25 (0.25 - 0.25, n=92) |
| Streptococcus pneumoniae | before | 0.125 (0.125 - 0.125, n=41) p=0.9811 |  |  | 0.063 (0.063 - 0.063, n=7) p=0.0098 |  |
|  | after | 0.125 (0.125 - 0.125, n=22) |  |  | 0.125 (0.063 - 0.125, n=9) |  |
| Streptococcus agalactiae | before | 0.125 (0.125 - 0.125, n=8) p=0.7771 |  | 0.125 (0.125 - 0.125, n=7) p=0.1392 | 0.125 (0.063 - 0.125, n=7) p=0.0044 |  |
|  | after | 0.125 (0.125 - 0.125, n=18) |  | 0.125 (0.125 - 0.125, n=18) | 0.125 (0.125 - 0.125, n=18) |  |
| Streptococcus oralis | before |  |  |  |  | 4 (2 - 8, n=24) p=0.7339 |
|  | after |  |  |  |  | 4 (1.25 - 8, n=7) |
| Enterococcus faecalis | before |  |  |  | 1 (0.5 - 1, n=284) p=0.9527 | 512 (512 - 512, n=277) p=0 |
|  | after |  |  |  | 1 (0.5 - 1, n=389) | 512 (8 - 512, n=391) |
| Enterococcus faecium | before |  |  |  | 16 (16 - 16, n=53) p=0 | 512 (512 - 512, n=52) p=5e-04 |
|  | after |  |  |  | 32 (32 - 32, n=39) | 4 (2 - 512, n=40) |
| Enterobacter cloacae | before | 1 (0.125 - 32, n=65) p=0.0352 | 1 (0.25 - 64, n=65) p=0.7483 | 0.125 (0.125 - 0.125, n=65) p=0.127 | 0.25 (0.125 - 0.5, n=65) p=0.29 | 0.25 (0.25 - 0.5, n=65) p=0.4569 |
|  | after | 0.25 (0.125 - 16, n=70) | 0.5 (0.25 - 64, n=70) | 0.125 (0.125 - 0.125, n=70) | 0.25 (0.25 - 0.5, n=70) | 0.5 (0.25 - 0.5, n=70) |
| Escherichia coli | before | 0.125 (0.125 - 0.125, n=554) p=0.7087 | 0.25 (0.25 - 0.25, n=554) p=0 | 0.125 (0.125 - 0.125, n=554) p=0 | 0.125 (0.125 - 0.125, n=554) p=0 | 0.5 (0.5 - 1, n=554) p=0.7773 |
|  | after | 0.125 (0.125 - 0.125, n=556) | 0.25 (0.25 - 0.5, n=556) | 0.125 (0.125 - 0.125, n=556) | 0.125 (0.125 - 0.125, n=556) | 0.5 (0.5 - 1, n=551) |
| Haemophilus influenzae | before | 0.125 (0.125 - 0.125, n=19) p=0.0388 |  |  |  |  |
|  | after | 0.125 (0.0785 - 0.125, n=22) |  |  |  |  |
| Haemophilus parainfluenzae | before | 0.125 (0.125 - 0.125, n=10) p=0.0865 |  |  | 0.25 (0.125 - 0.25, n=5) p=0.4376 |  |
|  | after | 0.125 (0.125 - 0.125, n=20) |  |  | 0.25 (0.25 - 0.5, n=9) |  |
| Klebsiella pneumoniae | before | 0.125 (0.125 - 0.125, n=84) p=0 | 0.25 (0.25 - 0.5, n=82) p=0.3017 | 0.125 (0.094 - 0.125, n=83) p=0 | 0.125 (0.125 - 0.25, n=84) p=0.2586 | 0.5 (0.25 - 0.5, n=84) p=0 |
|  | after | 0.125 (0.125 - 0.125, n=101) | 0.25 (0.25 - 0.5, n=101) | 0.125 (0.125 - 0.125, n=101) | 0.125 (0.125 - 0.25, n=101) | 0.25 (0.25 - 0.5, n=101) |
| Klebsiella oxytoca | before | 0.125 (0.125 - 0.4375, n=38) p=0.0752 | 0.25 (0.25 - 0.5, n=38) p=0.3126 | 0.125 (0.125 - 0.125, n=38) p=0.1615 | 0.125 (0.125 - 0.25, n=38) p=0.025 | 0.5 (0.25 - 0.875, n=38) p=0.0455 |
|  | after | 0.125 (0.125 - 0.125, n=66) | 0.25 (0.25 - 0.25, n=66) | 0.125 (0.125 - 0.125, n=66) | 0.25 (0.125 - 0.25, n=66) | 0.25 (0.25 - 0.5, n=66) |
| Acinetobacter baumanii | before | 8 (8 - 16, n=38) p=0.0144 | 4 (2 - 5, n=40) p=0.0241 | 0.25 (0.125 - 0.5, n=40) p=0.0017 | 0.125 (0.125 - 0.25, n=40) p=1e-04 | 0.5 (0.25 - 0.5, n=40) p=0.9272 |
|  | after | 16 (8 - 16, n=31) | 4 (4 - 8, n=31) | 0.5 (0.25 - 2, n=32) | 0.25 (0.25 - 0.25, n=32) | 0.5 (0.25 - 0.5, n=32) |
| Proteus mirabilis | before | 0.125 (0.125 - 0.125, n=74) p=0.0067 | 0.25 (0.25 - 0.25, n=73) p=0.5689 | 0.125 (0.125 - 0.125, n=74) p=0.9412 | 0.5 (0.25 - 2, n=74) p=0.6585 | 0.5 (0.25 - 1, n=74) p=0.6759 |
|  | after | 0.125 (0.125 - 0.125, n=103) | 0.25 (0.25 - 0.25, n=103) | 0.125 (0.125 - 0.125, n=103) | 0.25 (0.25 - 1.5, n=103) | 0.5 (0.5 - 0.5, n=103) |
| Serratia marcescens | before | 4 (0.125 - 8, n=80) p=0.0235 | 0.25 (0.25 - 0.5, n=80) p=0.8636 | 0.125 (0.125 - 0.125, n=80) p=0.0295 | 0.25 (0.125 - 0.25, n=80) p=0 | 0.5 (0.25 - 0.625, n=80) p=0.1425 |
|  | after | 0.5 (0.25 - 1, n=20) | 0.25 (0.25 - 0.5, n=20) | 0.125 (0.125 - 0.125, n=20) | 0.5 (0.5 - 1, n=20) | 0.5 (0.5 - 1, n=20) |
| Pseudomonas aeruginosa | before | 16 (8 - 32, n=400) p=0.4997 | 2 (1 - 4, n=398) p=0 | 0.5 (0.25 - 1, n=400) p=0 | 1 (1 - 2, n=400) p=0.5613 | 1 (1 - 2, n=400) p=0.6608 |
|  | after | 16 (16 - 16, n=185) | 4 (2 - 8, n=185) | 0.5 (0.25 - 2, n=185) | 1 (0.5 - 2, n=185) | 1 (1 - 4, n=185) |

| isolate | relocation | Vancomycin | Teicoplanin | Ciprofloxacin | Levofloxacin | Moxifloxacin |
| --- | --- | --- | --- | --- | --- | --- |
| Staphylococcus hominis | before | 1 (1 - 2, n=43) p=0.6044 | 0.25 (0.25 - 1.5, n=43) p=0.7789 | 2 (0.125 - 6, n=43) p=0.6098 | 1 (0.125 - 3, n=43) p=0.9051 | 0.094 (0.063 - 1, n=42) p=0.9712 |
|  | after | 1 (1 - 1, n=22) | 0.25 (0.25 - 1, n=22) | 0.625 (0.125 - 8, n=22) | 0.375 (0.0785 - 8, n=22) | 0.1875 (0.031 - 1.75, n=22) |
| Staphylococcus haemolyticus | before | 2 (1 - 2, n=32) p=0.9798 | 4 (2 - 8, n=31) p=0.0032 | 8 (8 - 8, n=32) p=0.5595 | 8 (2 - 8, n=31) p=0.8595 | 2 (1 - 2, n=31) p=0.7733 |
|  | after | 2 (1 - 3, n=15) | 8 (4 - 24, n=15) | 8 (8 - 8, n=15) | 4 (4 - 8, n=15) | 1 (1 - 4, n=15) |
| Staphylococcus epidermidis | before | 2 (1 - 2, n=218) p=0.4347 | 2 (1 - 4, n=219) p=0.7516 | 0.5 (0.25 - 8, n=218) p=0.0837 | 0.5 (0.25 - 4, n=219) p=0.5999 | 0.125 (0.063 - 1, n=219) p=0.0687 |
|  | after | 2 (2 - 2, n=86) | 2 (1 - 4, n=86) | 4 (0.25 - 8, n=82) | 4 (0.125 - 4, n=82) | 1 (0.063 - 2, n=85) |
| Staphylococcus aureus | before | 1 (1 - 1, n=177) p=0.8326 | 0.5 (0.25 - 1, n=179) p=0.034 | 0.25 (0.25 - 0.5, n=177) p=0.8005 | 0.25 (0.125 - 0.25, n=177) p=0 | 0.063 (0.031 - 0.063, n=173) p=0.3441 |
|  | after | 1 (1 - 1, n=93) | 0.5 (0.25 - 0.5, n=93) | 0.25 (0.25 - 0.5, n=90) | 0.125 (0.125 - 0.125, n=91) | 0.031 (0.031 - 0.063, n=93) |
| Streptococcus pneumoniae | before |  |  | 1 (0.875 - 2, n=8) p=0.8008 |  | 0.125 (0.063 - 0.125, n=32) p=0.2416 |
|  | after |  |  | 1 (1 - 2, n=9) |  | 0.125 (0.063 - 0.125, n=11) |
| Streptococcus agalactiae | before | 0.25 (0.25 - 0.5, n=7) p=0.0491 | 0.25 (0.25 - 0.25, n=7) p=0.1299 | 0.5 (0.5 - 0.75, n=7) p=0.777 | 0.5 (0.25 - 0.75, n=7) p=0.4567 | 0.125 (0.094 - 0.1875, n=7) p=0.4038 |
|  | after | 0.5 (0.5 - 0.5, n=18) | 0.25 (0.25 - 0.25, n=18) | 0.5 (0.5 - 0.5, n=18) | 0.5 (0.25 - 0.5, n=18) | 0.125 (0.063 - 0.125, n=21) |
| Streptococcus oralis | before |  |  |  |  | 0.125 (0.125 - 0.25, n=32) p=0.3442 |
|  | after |  |  |  |  | 0.125 (0.125 - 0.21875, n=14) |
| Enterococcus faecalis | before | 1 (1 - 2, n=283) p=0.8802 | 0.25 (0.25 - 0.25, n=282) p=1e-04 | 1 (1 - 2, n=283) p=0.0701 | 2 (1 - 2, n=283) p=0 | 0.25 (0.25 - 0.5, n=282) p=9e-04 |
|  | after | 1 (1 - 2, n=394) | 0.25 (0.25 - 0.25, n=394) | 1 (1 - 2, n=390) | 1 (0.5 - 1, n=374) | 0.25 (0.25 - 0.375, n=391) |
| Enterococcus faecium | before | 1 (0.5 - 1, n=53) p=0.0485 | 0.25 (0.25 - 0.5, n=53) p=0 | 4 (2 - 8, n=53) p=9e-04 | 4 (2 - 8, n=53) p=1e-04 | 2 (1 - 8, n=53) p=0.0012 |
|  | after | 1 (1 - 32, n=40) | 0.75 (0.5 - 11, n=40) | 8 (8 - 8, n=39) | 8 (8 - 8, n=39) | 8 (8 - 8, n=40) |
| Enterobacter cloacae | before |  |  | 0.031 (0.031 - 0.031, n=65) p=0.8102 | 0.031 (0.031 - 0.063, n=65) p=0.0168 | 0.063 (0.063 - 0.125, n=64) p=0.3198 |
|  | after |  |  | 0.031 (0.031 - 0.031, n=70) | 0.031 (0.031 - 0.063, n=70) | 0.063 (0.063 - 0.125, n=69) |
| Escherichia coli | before |  |  | 0.031 (0.031 - 0.031, n=554) p=0.01 | 0.031 (0.031 - 0.063, n=554) p=0.0013 | 0.031 (0.031 - 0.063, n=551) p=0 |
|  | after |  |  | 0.031 (0.031 - 0.031, n=556) | 0.031 (0.031 - 0.063, n=556) | 0.063 (0.031 - 0.125, n=553) |
| Haemophilus influenzae | before |  |  | 0.031 (0.031 - 0.031, n=26) p=0.1356 |  |  |
|  | after |  |  | 0.031 (0.031 - 0.055, n=22) |  |  |
| Haemophilus parainfluenzae | before |  |  | 0.125 (0.031 - 0.125, n=10) p=0.6912 |  |  |
|  | after |  |  | 0.125 (0.031 - 0.125, n=20) |  |  |
| Klebsiella pneumoniae | before |  |  | 0.031 (0.031 - 0.063, n=83) p=0.0693 | 0.063 (0.063 - 0.125, n=84) p=0 | 0.125 (0.063 - 0.125, n=83) p=0.4729 |
|  | after |  |  | 0.031 (0.031 - 0.031, n=101) | 0.031 (0.031 - 0.063, n=101) | 0.125 (0.063 - 0.125, n=101) |
| Klebsiella oxytoca | before |  |  | 0.031 (0.031 - 0.063, n=38) p=0.4699 | 0.063 (0.031 - 0.063, n=38) p=0.057 | 0.063 (0.063 - 0.125, n=38) p=0.3259 |
|  | after |  |  | 0.031 (0.031 - 0.055, n=66) | 0.031 (0.031 - 0.063, n=66) | 0.063 (0.063 - 0.125, n=66) |
| Acinetobacter baumanii | before |  |  | 0.125 (0.125 - 0.25, n=40) p=0.8632 | 0.125 (0.063 - 0.125, n=40) p=0.9331 | 0.063 (0.031 - 0.063, n=40) p=0.2243 |
|  | after |  |  | 0.1875 (0.125 - 0.3125, n=32) | 0.125 (0.063 - 0.25, n=32) | 0.063 (0.031 - 0.125, n=31) |
| Proteus mirabilis | before |  |  | 0.031 (0.031 - 0.063, n=74) p=1e-04 | 0.063 (0.063 - 0.063, n=74) p=0 | 0.25 (0.25 - 0.5, n=73) p=0.0176 |
|  | after |  |  | 0.031 (0.031 - 0.031, n=103) | 0.031 (0.031 - 0.063, n=103) | 0.25 (0.25 - 0.25, n=103) |
| Serratia marcescens | before |  |  | 0.063 (0.063 - 0.125, n=80) p=0.0117 | 0.125 (0.125 - 0.25, n=80) p=0.0024 | 0.25 (0.125 - 0.25, n=80) p=0.2405 |
|  | after |  |  | 0.063 (0.031 - 0.063, n=20) | 0.063 (0.031 - 0.125, n=20) | 0.25 (0.125 - 0.25, n=20) |
| Pseudomonas aeruginosa | before |  |  | 0.125 (0.125 - 0.25, n=400) p=0.3576 | 0.5 (0.25 - 1, n=400) p=0.1027 | 1 (1 - 4, n=399) p=0.9635 |
|  | after |  |  | 0.125 (0.125 - 0.5, n=185) | 0.5 (0.25 - 2, n=184) | 1 (1 - 4, n=181) |

| isolate | relocation | Clindamycin | Linezolid | Rifampicin | Cotrimoxazol | Colistin |
| --- | --- | --- | --- | --- | --- | --- |
| Staphylococcus hominis | before | 0.063 (0.063 - 0.125, n=42) p=0.8278 | 1 (0.5 - 1, n=40) p=0.9368 | 0.063 (0.063 - 0.063, n=43) p=0.0424 | 8 (1 - 64, n=43) p=0.8389 |  |
|  | after | 0.063 (0.063 - 0.4375, n=22) | 1 (0.5 - 1, n=22) | 0.063 (0.063 - 0.063, n=22) | 16 (4 - 32, n=21) |  |
| Staphylococcus haemolyticus | before | 0.125 (0.063 - 16, n=32) p=0.2363 | 1 (0.5 - 1, n=22) p=1 | 0.063 (0.063 - 0.063, n=32) p=0.0033 | 24 (8 - 256, n=32) p=0.0194 |  |
|  | after | 0.063 (0.063 - 0.625, n=15) | 1 (0.5 - 1, n=15) | 0.063 (0.047 - 0.063, n=15) | 16 (1 - 32, n=15) |  |
| Staphylococcus epidermidis | before | 0.125 (0.063 - 16, n=219) p=0.0129 | 0.5 (0.5 - 1, n=150) p=0.1911 | 0.063 (0.063 - 0.063, n=218) p=7e-04 | 8 (2 - 128, n=218) p=0 |  |
|  | after | 0.063 (0.063 - 3.5, n=86) | 0.5 (0.5 - 0.5, n=84) | 0.063 (0.063 - 0.063, n=86) | 4 (1 - 32, n=82) |  |
| Staphylococcus aureus | before | 0.063 (0.063 - 0.125, n=177) p=0 | 1 (1 - 1, n=111) p=0.978 | 0.063 (0.063 - 0.063, n=177) p=0 | 1 (1 - 1, n=176) p=0.0036 |  |
|  | after | 0.063 (0.063 - 0.063, n=93) | 1 (1 - 1, n=93) | 0.063 (0.063 - 0.063, n=93) | 1 (1 - 1, n=92) |  |
| Streptococcus pneumoniae | before | 0.063 (0.063 - 0.21875, n=12) p=0.1785 |  |  | 1 (1 - 2, n=5) p=0.6115 |  |
|  | after | 0.063 (0.063 - 0.063, n=10) |  |  | 2 (1 - 2, n=9) |  |
| Streptococcus agalactiae | before | 0.063 (0.063 - 0.0785, n=8) p=0.7418 | 0.75 (0.3125 - 1, n=6) p=0.6888 | 0.063 (0.063 - 0.063, n=7) p=0.4613 | 2 (2 - 4, n=7) p=0.1585 |  |
|  | after | 0.063 (0.063 - 0.063, n=21) | 0.75 (0.5 - 1, n=18) | 0.063 (0.063 - 0.063, n=18) | 1 (1 - 4, n=18) |  |
| Streptococcus oralis | before | 0.125 (0.063 - 0.125, n=46) p=0 |  |  |  |  |
|  | after | 0.063 (0.039 - 0.063, n=14) |  |  |  |  |
| Enterococcus faecalis | before | 16 (8 - 16, n=281) p=0 | 1 (1 - 2, n=233) p=6e-04 | 1.5 (1 - 4, n=282) p=0.0067 | 1 (1 - 1, n=279) p=0.004 |  |
|  | after | 8 (8 - 8, n=394) | 1 (1 - 2, n=395) | 1 (1 - 2, n=377) | 1 (1 - 1, n=385) |  |
| Enterococcus faecium | before | 4 (0.5 - 16, n=52) p=0.0596 | 1 (1 - 2, n=48) p=0.0017 | 4 (4 - 8, n=53) p=0.9211 | 16 (2 - 256, n=52) p=0.0673 |  |
|  | after | 8 (8 - 8, n=39) | 1 (0.5 - 1, n=40) | 8 (4 - 8, n=39) | 16 (1 - 32, n=38) |  |
| Enterobacter cloacae | before |  |  |  | 2 (2 - 4, n=65) p=0.0217 | 0.5 (0.5 - 1, n=59) p=0.9011 |
|  | after |  |  |  | 1.5 (1 - 4, n=70) | 0.5 (0.5 - 1, n=69) |
| Escherichia coli | before |  |  |  | 2 (1 - 256, n=553) p=0 | 0.5 (0.25 - 0.5, n=441) p=0.1502 |
|  | after |  |  |  | 1 (1 - 32, n=556) | 0.5 (0.25 - 0.5, n=552) |
| Haemophilus influenzae | before |  |  |  |  |  |
|  | after |  |  |  |  |  |
| Haemophilus parainfluenzae | before | 4 (2 - 4, n=5) p=0.1365 |  |  | 0.5 (0.5 - 0.5, n=5) p=0.0653 |  |
|  | after | 4 (4 - 12, n=11) |  |  | 1 (0.75 - 1, n=11) |  |
| Klebsiella pneumoniae | before |  |  |  | 4 (2 - 16, n=83) p=3e-04 |  |
|  | after |  |  |  | 1 (1 - 16, n=101) |  |
| Klebsiella oxytoca | before |  |  |  | 2 (1 - 7, n=38) p=0.053 |  |
|  | after |  |  |  | 1 (1 - 4, n=66) |  |
| Acinetobacter baumanii | before |  |  |  | 4 (2 - 4, n=40) p=0.0017 | 1 (0.5 - 2, n=32) p=0.0628 |
|  | after |  |  |  | 2 (1 - 2, n=32) | 0.5 (0.5 - 1, n=32) |
| Proteus mirabilis | before |  |  |  | 4 (2 - 208, n=74) p=0.0014 | 16 (16 - 16, n=58) p=0.1295 |
|  | after |  |  |  | 2 (1 - 16, n=103) | 16 (16 - 16, n=102) |
| Serratia marcescens | before |  |  |  | 8 (8 - 16, n=80) p=0 | 16 (16 - 16, n=65) p=0.3877 |
|  | after |  |  |  | 1 (1 - 1.75, n=20) | 16 (16 - 16, n=20) |
| Pseudomonas aeruginosa | before |  |  |  | 128 (64 - 256, n=378) p=0 | 1 (1 - 2, n=320) p=0.3188 |
|  | after |  |  |  | 16 (8 - 64, n=184) | 1 (1 - 2, n=183) |
